# Disruption of Glycogen Utilization Markedly Improves the Efficacy of Carboplatin against Preclinical Models of Clear Cell Ovarian Carcinoma

**DOI:** 10.3390/cancers12040869

**Published:** 2020-04-03

**Authors:** Tashbib Khan, Yaowu He, Thomas Kryza, Brittney S. Harrington, Jennifer H. Gunter, Mitchell A. Sullivan, Tahleesa Cuda, Rebecca Rogers, Claire M. Davies, Amy Broomfield, Madeline Gough, Andy C. Wu, Thomas McGann, S. John Weroha, Paul Haluska, Josephine M. Forbes, Jane E. Armes, Sinead C. Barry, Jermaine I. Coward, Nisha Jagasia, Naven Chetty, Cameron E. Snell, Rohan Lourie, Lewis C. Perrin, John D. Hooper

**Affiliations:** 1 Mater Research Institute, The University of Queensland, Translational Research Institute, Woolloongabba, QLD 4102, Australia; tashbib.khan@uq.net.au (T.K.); yaowu.he@mater.uq.edu.au (Y.H.); thomas.kryza@mater.uq.edu.au (T.K.); brittneyharr@gmail.com (B.S.H.); mitchell.sullivan@mater.uq.edu.au (M.A.S.); tahleesa.cuda@gmail.com (T.C.); rebecca.rogers@mater.uq.edu.au (R.R.); TR-ANZGOG@anzgog.org.au (C.M.D.); andy.wu@tri.edu.au (A.C.W.); thomas.p.mcgann95@gmail.com (T.M.); josephine.forbes@mater.uq.edu.au (J.M.F.); jane.armes@health.qld.gov.au (J.E.A.); sineadcbarry@gmail.com (S.C.B.); jim.coward@gmail.com (J.I.C.); Cameron.Snell@mater.org.au (C.E.S.); Rohan.Lourie@mater.org.au (R.L.); lewisperrin@mc.mater.org.au (L.C.P.); 2 Australian Prostate Cancer Research Centre-Queensland, Institute of Health and Biomedical Innovation, School of Biomedical Sciences, Faculty of Health, Queensland University of Technology, Translational Research Institute, Brisbane, QLD 4102, Australia; jennifer.gunter@qut.edu.au; 3 Mater Brisbane Hospital, Mater Health Services, South Brisbane, QLD 4101, Australia; amy.broomfield@health.qld.gov.au (A.B.); Madeline.Gough@mater.org.au (M.G.); Nisha.Jagasia@mater.org.au (N.J.); Naven.Chetty@mater.org.au (N.C.); 4 Department of Medical Oncology, Mayo Clinic, Rochester, MN 55905, USA; weroha.saravut@mayo.edu (S.J.W.); paul.haluska@bms.com (P.H.); 5 Bristol-Myers Squibb, Princeton, NJ 08540, USA; 6 ICON Cancer Care, South Brisbane, QLD 4101, Australia

**Keywords:** 2DG, glycogen, clear cell ovarian cancer

## Abstract

High stage and recurrent ovarian clear cell carcinoma (OCC) are associated with poor prognosis and resistance to chemotherapy. A distinguishing histological feature of OCC is abundant cytoplasmic stores of glucose, in the form of glycogen, that can be mobilized for cellular metabolism. Here, we report the effect on preclinical models of OCC of disrupting glycogen utilization using the glucose analogue 2-deoxy-D-glucose (2DG). At concentrations significantly lower than previously reported for other cancers, 2DG markedly improves the efficacy in vitro of carboplatin chemotherapy against chemo-sensitive TOV21G and chemo-resistant OVTOKO OCC cell lines, and this is accompanied by the depletion of glycogen. Of note, 2DG doses—of more than 10-fold lower than previously reported for other cancers—significantly improve the efficacy of carboplatin against cell line and patient-derived xenograft models in mice that mimic the chemo-responsiveness of OCC. These findings are encouraging, in that 2DG doses, which are substantially lower than previously reported to cause adverse events in cancer patients, can safely and significantly improve the efficacy of carboplatin against OCC. Our results thus justify clinical trials to evaluate whether low dose 2DG improves the efficacy of carboplatin in OCC patients.

## 1. Introduction

Ovarian clear cell carcinoma (OCC) is the second most common type of epithelial ovarian cancer (EOC), comprising 5–25% of all ovarian cancers, depending on ethnicity [1,2]. Patients are managed for the other EOC histotypes with cytoreductive surgery and chemotherapy [3,4]. However, the platinum-based chemotherapy that is applied to other histotypes, such as the more common high-grade serous ovarian carcinoma (HGSOC), is often ineffective for OCC [4,5], with recurrent and high-stage disease particularly resistant to this treatment [6,7,8,9]. The paucity of treatment options is reflected in the poorer prognosis of OCC compared to other EOC histotypes [7,10].

OCCs are molecularly and histologically distinct from other EOCs, with several features thought to mediate resistance to conventional therapies [11]. This includes abundant cytoplasmic stores of glucose in the form of glycogen that, when metabolized, protect cancer cells from stressors such as chemotoxic agents, low oxygen levels, and nutrient deficiency [9]. Glycogen causes the clear cytoplasmic appearance that is a conspicuous histological feature of OCC [12]. The biological importance of the characteristic storage of glucose and its release from glycogen is further highlighted by the observation that 18F-fluoro-deoxy-D-glucose positron-emission tomography, which relies on tumor glycolysis for its efficacy, is potentially prognostic for OCC [13]. 

In the present work, we sought to target the reliance of OCC on glycogen using the glucose analogue 2-deoxy-D-glucose (2DG), evaluating its impact on the efficacy of the platinum chemotherapy most commonly used for ovarian cancer, carboplatin. 2DG competitively inhibits cellular utilization of glucose by several mechanisms. Unlike glucose, 2DG cannot undergo isomerization at its second carbon, which is required to generate fructose 6-phosphate, an essential intermediary feeding into glycolysis, and the pentose phosphate pathway for energy production and generation of the biosynthetic material required for cell proliferation [14,15,16]. In addition, 2DG can competitively inhibit glucose uptake by cells via glucose transporters, as well as inhibiting hexokinase, the initiating enzyme in glucose metabolism [14,15]. In addition, at the elevated concentrations commonly employed in in vitro models, 2DG can elicit anti-cancer effects in normoxic conditions by inhibiting N-linked glycosylation to induce endoplasmic-reticulum (ER) stress and the mis-folded protein response [17]. In fact, the inhibitory effects of 2DG at high concentration at or above 5 mM are well known to improve the efficacy of a range of agents against cancer cells in vitro [18]. For example, two HGSOC cell lines and malignant cells isolated from the ascites of 17 HGSOC patients, had markedly improved in vitro responses to cisplatin and carboplatin when these agents were combined with 5 or 10 mM 2DG [19]. Similar and higher concentrations of 2DG improved the responses in vitro of a range of breast cancer cell lines to mitochondria targeted drugs [20,21], a glucopyranoside [22], and inhibitors of BCL family members [23]. Analogous in vitro data have been reported for cervical cancer [24], melanoma [25], glioblastoma [26], and osteosarcoma [27]. In the only study targeting a clear cell pathology, 5 mM 2DG reduced proliferation and viability of primary cultures of renal clear cell carcinoma cells [18].

Of note, these elevated 2DG concentrations employed in vitro are well above the maximum plasma levels (C_max_) achieved in cancer patients in two published dose-finding clinical trials. In a study of patients with advanced solid malignancies, the maximum tolerated dose for 2DG, administered daily during the first two weeks of a three week cycle, was 45 mg/kg, resulting in a C_max_ 0.449 mM [28], which is 10-fold lower than concentrations typically employed in in vitro studies [18,19,20,21,22,23,24,25,26,27]. In a combination trial with docetaxel for a range of advanced solid tumors, the clinically tolerable dose for 2DG, administered daily during the first and third weeks of a four week cycle during the dose-escalation phase, was 63 mg/kg/day, resulting in a C_max_ of 0.707 mM [29]. Significant adverse events during these studies included cardiac and gastrointestinal toxicities and neutropenia [28,29], and one patient had a fatal cardiac arrest 17 days after the last dose, which was considered to be related to 2DG [29]. 

Here we have examined for the first time the effects on OCC of disrupting glucose metabolism using 2DG in combination with carboplatin. We were particularly interested in whether physiologically achievable levels of 2DG could disrupt the in vitro and in vivo growth of this cancer, which is reliant on large cytoplasmic stores of glucose as a protective mechanism against stressors, including chemotherapy. We report that low levels of 2DG significantly improve the efficacy of carboplatin against chemo-sensitive and chemo-resistant OCC cell lines in vitro, and OCC xenograft and patient-derived models in vivo.

## 2. Results

### 2.1. Effect of 2DG on In Vitro Growth of OCC Cell Lines with Distinct Chemo-Sensitivities

**Figure 1:**
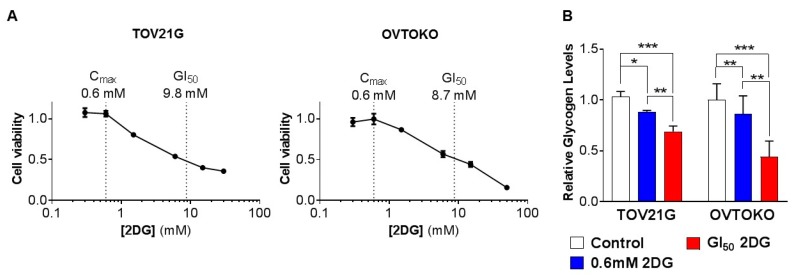
Effect of 2-deoxy-D-glucose (2DG) on in vitro growth of ovarian clear cell carcinoma (OCC) cell lines with distinct chemo-sensitivities. TOV21G and OVTOKO cells were treated with increasing concentrations of 2DG (TOV21G cells 0–30 mM; OVTOKO cells 0–50 mM) for 72 h. (**A**) Cell viability was quantified using a CellTiter assay kit and a PHERAstar Omega plate reader. The calculated GI_50_ value for each line is shown as well as an average of the 2DG C_max_ value achieved in cancer patients [1,2]. (**B**) Graphs of relative glycogen levels quantified from cells collected after a 72 h treatment period. Results are presented as mean +/− SEM from triplicates of three independent assays. *, *p* ≤ 0.05; **, *p* ≤ 0.01; ***, *p* ≤ 0.001.

We first assessed the impact of inhibiting glucose utilization on the viability of OCC cells by treating the chemo-sensitive OCC cell line TOV21G and the chemo-resistant OCC cell line OVTOKO with increasing concentrations of 2DG. After 72 h of treatment, the 2DG concentration that caused a 50% reduction in cell viability (GI_50_) was 9.8 mM for TOV21G cells (Figure 1A, left) and 8.7 mM for OVTOKO cells (Figure 1A, right). These values compared well with the GI_50_ values published for the HGSOC cell lines CaOv4 and SKOV3 of 14 mM and 18 mM, respectively [19]. Of note, the GI_50_ values for TOV21G and OVTOKO cells were more than 10 times greater than the C_max_ values of 0.449 mM and 0.707 mM achieved in cancer patients [28,29]. A physiologically achievable concentration of 2DG of 0.6 mM, which lies between these C_max_ values, was accompanied by a small decrease of ~15% in relative glycogen content in each cell line, while treatment at the GI_50_ concentration resulted in reductions in glycogen of ~35% for TOV21G and ~55% for OVTOKO cells (Figure 1B).

**Figure 2:**
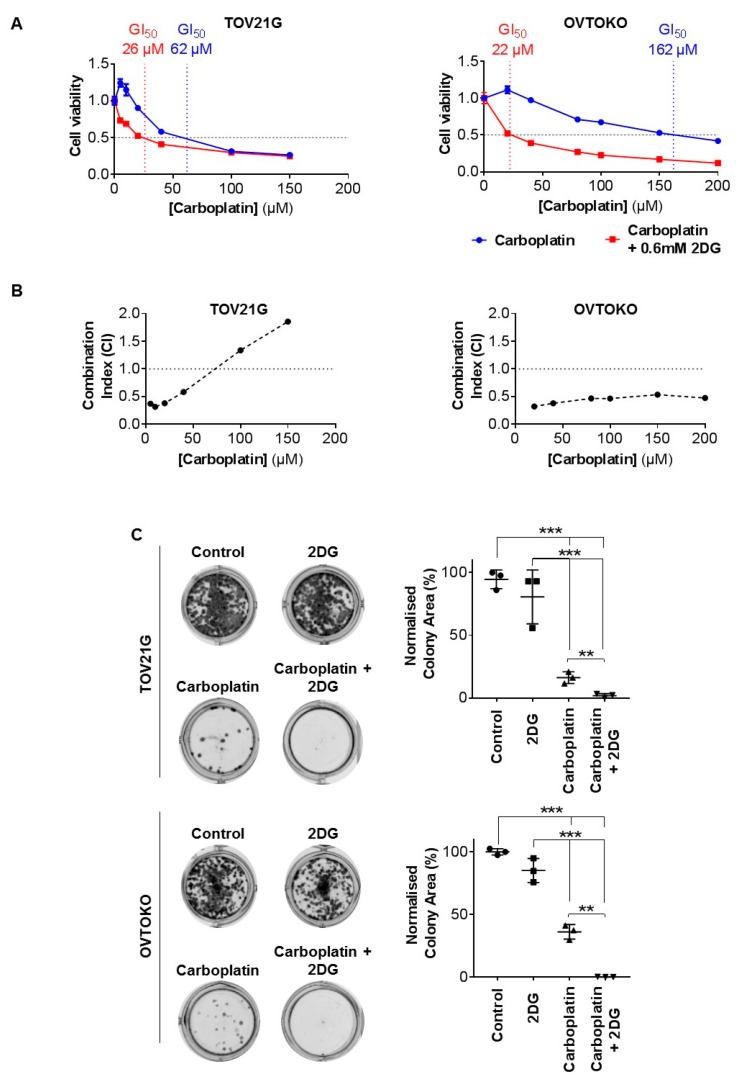
Here, 2DG at a physiologically achievable concentration acts synergistically with carboplatin against OCC cells in vitro. (**A**) TOV21G (left) and OVTOKO (right) cells seeded in 96 well plates overnight were treated for 72 h with carboplatin (TOV21G cells 0–150 μM; OVTOKO cells 0–200 μM) or carboplatin combined with 2DG (0.6 mM). Cell viability was quantified and graphed to determine GI_50_ (blue, carboplatin; red, carboplatin combined with 2DG). (**B**) Synergism was assessed in TOV21G (left) and OVTOKO (right) cells using the Chou-Talalay Combination Index (CI) method. CI < 1 indicated synergism. (**C**) Colony formation assays were performed on TOV21G (upper) and OVTOKO (lower) cells plated overnight in six well plates then treated with phosphate buffer saline (PBS), 2DG (0.6 mM), carboplatin (GI_50_ concentration determined in A), or carboplatin combined with 2DG. After 72 h cells were lifted then seeded at low density in antibiotic-free media for 10 days. Established cell colonies were then stained with crystal violet, imaged (left, representative images) and signal quantified using ImageJ software and normalized to PBS treated cells with the results graphed (right). Results are presented as mean +/− SEM from triplicates of three independent assays. **, *p* ≤ 0.01; ***, *p* ≤ 0.001.

To examine the impact of targeting glucose metabolism, using a physiologically achievable concentration, on the efficacy of carboplatin against OCC, we employed 2DG in vitro at 0.6 mM. Whereas 2DG at this concentration as a single agent had no impact on the viability of TOV21G and OVTOKO cells (Figure 1A), it significantly reduced the viability of these cells when combined with carboplatin. As shown in Figure 2A, 0.6 mM 2DG more than halved the GI_50_ of carboplatin against chemo-sensitive TOV21G cells from 62 µM to 26 µM, and even more impressively, reduced the GI_50_ against chemo-resistant OVTOKO cells by ~86% from 162 µM to 22 µM. Assessment of interactions between the two agents using the Chou-Talalay combination index method, which assesses for synergism, additive effects, and antagonism between agents used in combination [30], indicated that 2DG acts synergistically on TOV21G cells up to ~65 µM of carboplatin, with synergism apparent against OVTOKO cells across the whole concentration range tested for carboplatin up to ~200 µM (Figure 2B). To further examine the effect of 2DG on the efficacy of carboplatin, we used a clonogenic survival assay. As expected, carboplatin as a single agent reduced colony formation, and we noted once again that although 0.6 mM 2DG had little impact on colony formation, its combination with carboplatin (at GI_50_ concentration) significantly reduced colony forming capacity, particularly of chemo-resistant OVTOKO cells (Figure 2C). 

### 2.2. 2DG in Combination with Carboplatin Reduces Glycogen Levels in OCC Cells In Vitro

**Figure 3:**
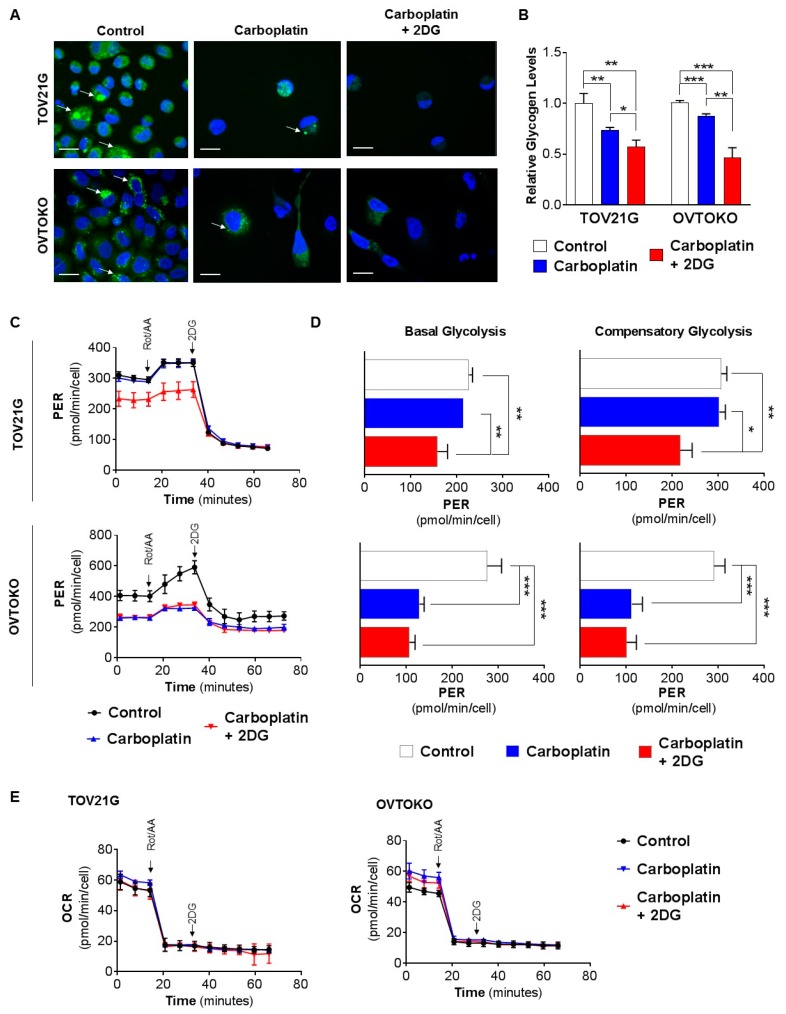
In combination with carboplatin, 2DG reduces glycogen levels in OCC cells in vitro. (**A**,**B**) Cells were treated for 72 h with PBS, carboplatin (GI_50_ concentration determined in Figure 2A), or carboplatin and 2DG (0.6 mM). (**A**) Confocal microscope images of cells incubated with green fluorescent 2-NBDG for 3 h. Carboplatin and carboplatin/2DG treatments considerably reduced cell numbers, which was accompanied by reduced levels of 2-NBDG fluorescent signal per cell. Scale bar, 20 µm. (**B**) Graphs of relative glycogen levels quantified by analysis of lysates from cells collected prior to 2-NBDG treatments. (**C**) Graph of PER for TOV21G (upper) and OVTOKO (lower) cells treated with PBS, as control, carboplatin (GI_50_ concentration determined in Figure 2A), or carboplatin, combined with 2DG (0.6 mM) for 24 h. (**D**) Graph of basal (left) and compensatory (right) PER for TOV21G (upper) and OVTOKO (lower) cells. Data were derived from (C). (**E**) Graph of OCR for TOV21G (left) and OVTOKO (right) cells treated with PBS, as control, carboplatin (GI_50_ concentration determined in Figure 2A), or carboplatin combined with 2DG (0.6 mM) for 24 h. In C to E, the inhibitors rotenone and antimycin A were introduced to eliminate oxidative phosphorylation, followed by 2DG to ablate glycolysis. Results are presented as mean +/− SEM from triplicates of three independent assays. *, *p* ≤ 0.05; **, *p* ≤ 0.01; ***, *p* ≤ 0.001.

To examine glycogen levels, we first performed confocal microscopy analysis of the signal from green fluorescent 2-NBDG. This agent, which labels glycogen in living cells [31], was incorporated in the two OCC cell lines in response to treatments for 72 h with carboplatin (GI_50_ concentration), or 2DG (0.6 mM) combined with carboplatin, in comparison with phosphate buffer saline (PBS). Both carboplatin and combined 2DG and carboplatin considerably reduced the number of attached TOV21G and OVTOKO cells, and this was accompanied by reduced levels of 2-NBDG fluorescence per cell, consistent with a loss of glycogen content (Figure 3A). These data were supported by a quantitative measurement of D-glucose levels released by an amyloglucosidase degradation of glycogen that remained in these lines after the treatments. Carboplatin as a single agent reduced relative glycogen levels by ~30% in chemo-sensitive TOV21G cells and ~15% in chemo-resistant OVTOKO cells, and levels were reduced by ~45% and ~60% in these lines, respectively, when 2DG was combined with carboplatin (Figure 3B). 

We next assessed the effect of 2DG on carboplatin-induced changes in glucose metabolism to produce energy via cellular respiration involving glycolysis and the citric-acid (TCA) cycle, the two major energy-producing pathways in mammalian cells. Impacts on glycolysis were assessed by measuring changes in extracellular acidification rate (ECAR), and effects on the citric acid cycle were examined by measuring changes in oxygen consumption rate (OCR). The assay format we employed measures these parameters simultaneously to arrive at a total cellular proton efflux rate (PER; acidification is due to the combined effects of glycolysis and the TCA cycle), and a glycolytic acidification rate due solely to glycolytic acidification, which is derived by subtracting TCA cycle-derived acidification from PER [32]. Basal glycolysis is the steady-state PER, prior to the addition of the oxidative phosphorylation inhibitors rotenone and antimycin A (Figure 3C). Introduction of these inhibitors into the experimental system eliminates mitochondrial ATP production, permitting calculation of TCA cycle-derived PER. The inhibitors force cells to increase glycolysis for energy production, a state known as compensatory glycolysis. Introduction of 2DG then inhibits glycolysis and establishes the extent to which the PER value is due to cellular processes other than glycolysis and TCA cycle (Figure 3C). In these assays, both cell lines displayed significantly reduced basal and compensatory glycolysis in response to the 2DG/carboplatin combination reducing by ~30% in TOV21G cells (upper) and ~60% in OVTOKO (lower) cells (Figure 3D, compare blue and black lines). Of note, there were significant differences between the two OCC cell lines in response to carboplatin alone. While carboplatin as a single agent had no impact on basal and compensatory glycolysis of chemo-sensitive TOV21G cells (Figure 3D, upper, compare blue and black lines), both these parameters were reduced by ~60% in chemo-resistant OVTOKO cells in response to carboplatin (Figure 3D, lower, compare blue and black lines). 

The effects on the TCA cycle, assessed by measuring changes in OCR quantifying oxygen concentration, indicated that the cycle was unaffected in both OCC cell types by carboplatin or the 2DG/carboplatin combination (Figure 3E). Together, these findings indicate that the observed increased glycogen consumption by OCC cells in vitro in response to combined 2DG and carboplatin is not due to increased glycolysis or oxidative phosphorylation. 

### 2.3. 2DG Combined with Carboplatin Reduces Tumor Burden of OCC Xenografts in Mice

To test the impact of 2DG on the efficacy of carboplatin against OCC in vivo, we employed a xenograft model, using chemo-sensitive TOV21G cells, and three PDX models in mice. To mimic disseminated disease in patients, for these experiments OCC cells were injected intraperitoneally (i.p.), and treatments began two weeks later, when a large number of tumor nodules have typically developed in the peritoneal cavity for this ovarian cancer histotype [7]. Because we were conscious of the adverse events that can accompany use of 2DG in humans [28,29], we employed this agent at 50 mg/kg/week for the cell line model and 50 mg/kg twice weekly for the PDX models. These are less than one-tenth of the previously reported dose of 500 mg/kg/day for an OCC cell line xenograft model [33], and considerably lower than those administered to cancer patients when these are converted to a mouse equivalent dose (MED), using an established conversion factor [34]. Using this factor, the MED for the clinical trial employing a 2DG dose of 45 mg/kg/day [28] is 553.5 mg/kg/day, and for the second trial employing a dose of 63 mg/kg/day [29], the MED is 774.9 mg/kg/day.

**Figure 4:**
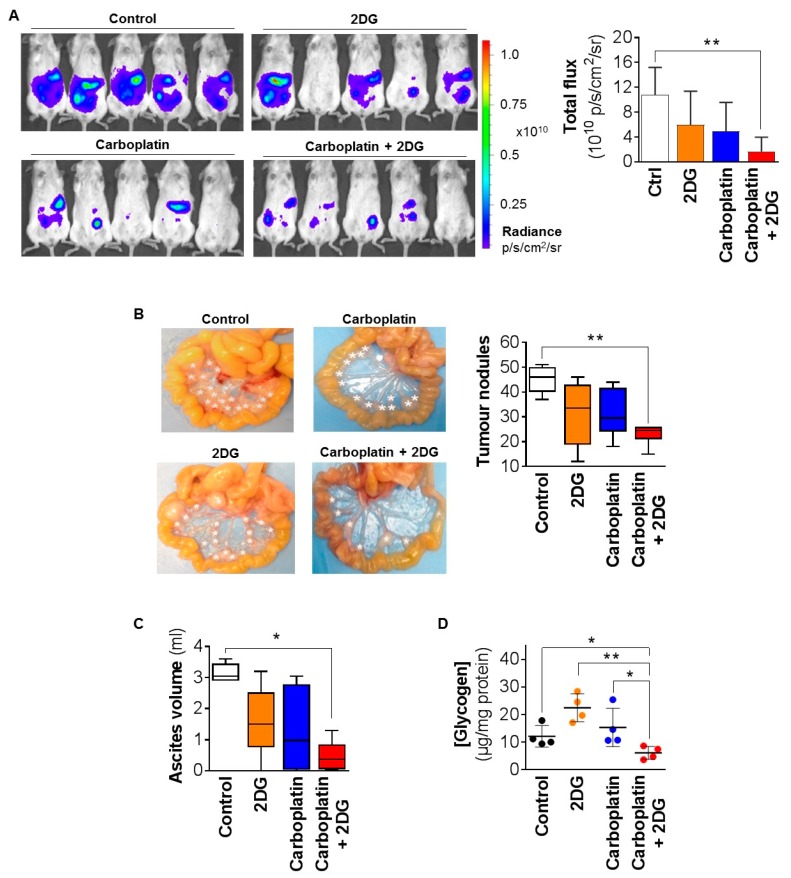
Combined with carboplatin, 2DG reduces the tumor burden of TOV21G cell line xenografts. Luciferase-labelled TOV21G cells (2 × 10^5^) were injected intraperitoneally (i.p.) into female NSG mice, with treatment starting two weeks later with PBS (control), 2DG (50 mg/kg/week), carboplatin (10 mg/kg/week), or a combination 2DG and carboplatin. Bioluminescent tumor burden was measured weekly with mice sacrificed at day 37. (**A**) Left, representative bioluminescent images of mice. Luminescence signal is provided in radiance units (p/s/cm2/sr). Right, graph of bioluminescence signal. (**B**) Left, representative images of mesenteric membrane of mice harvested from each group, with tumor nodules indicated by white asterisks. Right, graph of tumor nodules ≥ 8 mm^3^. (**C**) Graph of ascites volume. (**D**) Graph of glycogen content of tumors. Results are presented as mean +/− SD. *, *p* ≤ 0.05; **, *p* ≤ 0.01.

Quantitative analysis of bioluminescent imaging of mice carrying xenografts of luciferase-labelled TOV21G cells (Figure 4A, left) revealed that, while 2DG and carboplatin as single agents did not significantly reduce the signal from tumors, a significant reduction of ~75% was apparent from the combination of these agents compared with control treated mice (Figure 4A, right). Consistent data were obtained by quantifying tumor burden, which was apparent as distinct nodules, throughout the abdominal cavity, and ascites. Images of the mesenteric membrane from the mice highlighted the significant numbers of tumor nodules in control-treated mice, mimicking the disseminated disease generally encountered in patients (Figure 4B, left). PBS-treated mice had an average of ~46 nodules per mouse, and 2DG combined with carboplatin caused a statistically significant reduction to ~24 nodules (Figure 4B, right). There was a trend towards the single agents also reducing the tumor burden, however, the data did not reach statistical significance (~33 and ~30 nodules in response to 2DG and carboplatin, respectively; Figure 4B, right). Similar effects were observed for ascites which, in PBS-treated mice, was an average of ~3 mL, with a statistically significant reduction to ~0.4 mL in response to the 2DG/carboplatin combination, with the results for the single agents not reaching statistical significance (Figure 4C). There were no differences between treatment groups in terms of general health or body weight, indicating that 2DG at the employed dose had no toxic effects. The effects on tumor burden were accompanied by altered glycogen content in xenografts. The average content of ~12 µg/mg/protein in PBS treated mice reduced to ~6 µg/mg/protein, in response to the 2DG/carboplatin combination (Figure 4D). There was also a trend towards increased levels of glycogen to 23 µg/mg/protein in response to 2DG as a single agent, but this result did not reach statistical significance (Figure 4D). 

**Figure 5:**
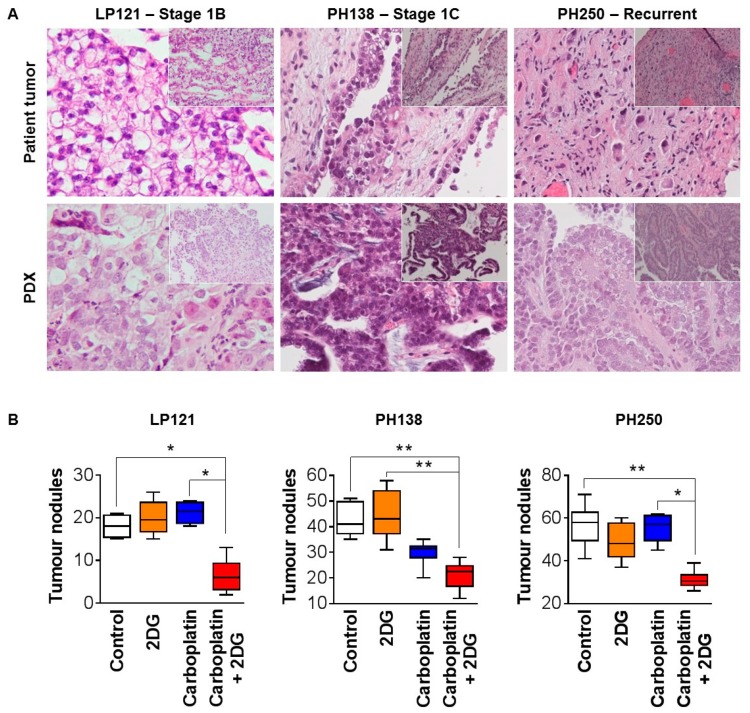
Combined with carboplatin, 2DG reduces tumor burden of PDX models of OCC. (**A**) Images of hematoxylin and eosin stained sections of representative tumors from patient LP121, PH138 and PH250, and PDX models developed from these tumors. Morphological features of patient tumors are retained in the corresponding PDX. (**B**) Graph of tumor nodules ≥8 mm^3^. For each model cell, slurry was injected i.p. into female NSG mice. After two weeks of growth, tumors were treated with PBS (control), 2DG (50 mg/kg twice weekly), carboplatin (1 mg/kg/week), or 2DG and carboplatin. Treatments continued until control mice reached ethical endpoint, when all mice were euthanized, and tumor burden quantified by counting tumor nodules ≥ 8 mm^3^. Results are presented as mean +/− SD. *, *p* ≤ 0.05; **, *p* ≤ 0.01.

Data from PDX models were consistent with findings from the xenograft model that 2DG markedly improves the efficacy of carboplatin against OCC in vivo. For these assays, we employed two models derived from low stage tumors resected prior to chemotherapy (LP121 and PH138), and a third model derived from a recurrent tumor (PH250). The models displayed histological features consistent with the tumor, from which each was derived (Figure 5A). As was observed for TOV21G cell line xenografts, PDX tumor burden was apparent as distinct nodules, throughout the abdominal cavity, although only one model (PH250) had ascites, and the volume was small. The tumor burden of the PDXs was unaffected by single agent 2DG, and both LP121 and PH250 were unresponsive to carboplatin, while PH138 displayed sensitivity to this chemotherapy, with the average number of tumor nodules per mouse reducing from ~41 to ~30 (Figure 5B). Combined 2DG and carboplatin caused marked statistically significant reductions, compared with control treatment, from ~18 to ~6 nodules for chemo-resistant LP121, from ~41 to ~21 nodules for chemo-sensitive PH138, and ~59 to ~30 nodules for chemo-resistant PH250 (Figure 5B). Of the PDX models, only PH250 accumulated ascites, and the volumes for this model were low, with no statistically significant changes in response to the treatments. There were no differences between treatment groups in terms of general health or body weight, indicating that 2DG at the employed dose had no toxic effects. 

## 3. Discussion

The key finding from this study is that low levels of the glucose analogue 2DG markedly improve the efficacy of carboplatin against preclinical models of OCC. Our results support future clinical trials to evaluate whether low dose 2DG can similarly improve the efficacy of carboplatin in OCC patients.

Importantly, the 2DG doses employed by us in vivo against cell line and PDX mouse models were several orders of magnitude lower than those used in two reported dose-finding clinical studies in cancer patients. The 2DG doses in the patient studies, 45 mg/kg/day during week one and two of a three week cycle [28], and 63 mg/kg/day during the first and third weeks of a four week cycle [29], were accompanied by significant adverse events, including cardiac and gastrointestinal toxicities and neutropenia [28,29], with one patient dying from 2DG-associated cardiac arrest 17 days after the last dose [29]. To avoid these adverse events, we employed 2DG at 50 mg/kg/week against an OCC cell line xenograft model, and at 50 mg/kg twice weekly against three PDX models. Using an established conversion factor [35] these doses are more than 10-fold lower than the equivalent doses derived from the two human studies. The human dose of 45 mg/kg/day is equivalent to 553.5 mg/kg/day in mice, while the human dose of 63 mg/kg/day equates to 774.9 mg/kg/day in mice. 

Employing these significantly reduced 2DG doses, data from a TOV21G cell line xenograft model showed impressive 2DG/carboplatin-induced reductions in tumor burden from ~46 to ~24 tumor nodules, and ~3 mL to ~0.4 mL of ascites, whereas carboplatin as a single agent failed to have a statistically significant impact on these parameters. Similarly, impressive results were obtained from the three PDX models. Because TOV21G cells are chemo-sensitive, we designed the PDX assays to address the key clinical issue of OCC resistance to carboplatin, so that two of the PDXs were largely resistant, and one sensitive to the carboplatin dose were employed in these in vivo experiments. LP121, derived from a low stage OCC, and PH250, derived from a recurrence, were resistant to carboplatin, while PH138, derived from another low stage OCC, was sensitive to carboplatin. Of note, all three models displayed significantly increased sensitivity to carboplatin in the presence of low dose 2DG; compared with control treated mice LP121, tumor burden reduced by 66%, while PH138 and PH250 reduced by ~50%. These findings, which were obtained using a 2DG dose designed primarily to avoid toxicities, justify dose escalation studies in patients to determine an optimal dose against OCC.

Consistent with reports showing important contributions of glycogen to cancer cell proliferation, survival, and protection [35,36], our results suggest that this distinguishing histological feature of OCC likely mediates the ability of 2DG to increase the efficacy of carboplatin against this cancer. Our findings indicate that 2DG acts synergistically, with carboplatin to markedly reduce the viability of OCC cells in vitro by promoting apoptosis. While low concentrations of 2DG as a single agent have little impact on glycogen levels, combination with carboplatin significantly reduces OCC glycogen levels particularly in chemo-resistant OVTOKO cells. This effect was also seen in vivo in TOV21G cell xenografts, which were significantly depleted of glycogen in response to combined low dose 2DG and carboplatin. 

The mechanism by which glycogen depletion is promoted by the 2DG/carboplatin combination is not yet clear, but is potentially via effects on glycogen synthesis, rather than on glycogen consumption. This is because our data, showing that the combination did not increase PER above basal levels in TOV21G or OVTOKO cells, indicates that the observed glycogen depletion is not due to increased rates of energy consumption via glycolysis or oxidative phosphorylation. Instead, the findings suggest the possibility that glycogen depletion occurs because 2DG/carboplatin disrupts glycogen synthesis. It should also be noted that the level of residual glycolysis after 2DG treatments continue to contribute to glycolysis. For both cell lines in our in vitro models, this level was significant, ~70% residual glycolysis for TOV21G and 40% for OCTOKO cells. Furthermore, lactate production should also ideally be quantified as a second measure of glycolysis, in addition to extracellular acidification. To understand the mechanism by which glycogen is depleted in response to 2DG/carboplatin, further research is required to quantify the effect of this combination on glycogen synthesis, including the rate of glucose transport across the plasma membrane, and the rate of glucose incorporation into glycogen, as well as glycogen consumption including incorporation of metabolites into lipid biosynthesis. 

The glycogen depletion observed by us in OCC models potentially provides important insight into why non-clear cell types of cancer typically require several orders of magnitude higher levels of 2DG to significantly improve the efficacy of therapeutic agents. These other cancers include HGSOC [19], breast cancer [20,21,22,23], cervical cancer [24], melanoma [25], glioblastoma [26], and osteosarcoma [27], which typically require 2DG concentrations ≥ 5 mM to increase the efficacy in vitro of chemotherapies and other therapeutic agents. This contrasts with our data, which show that 0.6 mM 2DG is sufficient to significantly improve the efficacy of carboplatin against OCC cells in vitro. It is possible that the lack of stored glucose in these non-clear cell cancers, which causes reliance on extracellular glucose for energy, requires much higher levels of 2DG to improve the efficacy of therapeutic agents compared with glycogen containing malignancies like OCC.

## 4. Materials and Methods 

### 4.1. Reagents and Antibodies

2DG powder was purchased from Sigma-Aldrich (Castle Hill, Australia) and dissolved in UltraPure Distilled Water (Thermo Scientific, Carlsbad, CA, USA). Carboplatin was obtained from Mater Pharmacy Services (Mater Adult Hospital, South Brisbane, Australia) and diluted in sterile saline (Pfizer, New York, NY, USA) for mouse experiments. All other reagents were from Sigma-Aldrich except where noted.

### 4.2. Cell Lines and Culture Conditions

The chemo-sensitive OCC cell line TOV21G was generously provided by Dr Katherine Roby (University of Kansas School of Medicine, Kansas City, KS, USA), and the chemo-resistant OCC cell line OVTOKO by Professor Gottfried Konechny (UCLA Medical Centre, University of California, Los Angeles, CA, USA). TOV21G cells were maintained in a 1:1 mixture of MCDB 105 and Medium 199, supplemented with 15% fetal bovine serum (FBS; Thermo Scientific). OVTOKO cells were maintained in RPMI medium containing glutamine and 10% FBS. Both lines were cultured in media supplemented with penicillin (100 units/mL) and streptomycin (100 units/mL), and maintained at 37 °C in a humidified 5% CO2 atmosphere [7]. Cells were passaged at 60–70% confluency in non-enzymatic dissociation buffer, containing 0.5 mM ethylene diamine-tetra-acetate (EDTA) in phosphate buffer saline (PBS). Cell lines were cultured for less than three months, after which time a fresh aliquot was revived from liquid nitrogen storage for culture.

### 4.3. Luciferase Labelling of Cells

TOV21G and OVTOKO cells were labelled with a lentivirus-based expression construct generated by cloning a polymerase chain reaction (PCR)-amplified DNA fragment encoding luciferase from a pGL4.10-lucIIplasmid (Promega, Sydney, Australia) into a pLenti CMV Hygro DEST vector (Addgene, Cambridge, MA, USA) using Gateway LR recombination cloning technology (Life Technologies, Mulgrave, Australia), as previously described [7]. Cells stably transduced with the luciferase construct were selected in puromycin (2 μg/mL; Thermo Scientific) [7,37] then imaged to examine morphology using an Olympus Inverted IX73 microscope and associated cellSens standard 1.7 imaging software (Olympus, Notting Hill, Australia) [38]. Luciferase labelling was also assessed by plating cells in duplicate in serial dilutions from 40,000 cells/well and measuring luminescence on an IVIS Bioluminescence System, using the associated LivingImage software (Caliper Life Sciences, Mountain View, CA, USA) as described previously [7].

### 4.4. Cell Viability Assay

Cells (1500 cells/well) were plated in triplicate in 96 well plates and allowed to attach overnight, before treatment for 72 h with 2DG (TOV21G cells 0–30 mM; OVTOKO cells 0–50 mM). In a subsequent experiment cells similarly plated were treated with carboplatin (TOV21G cells 0–150 μM; OVTOKO cells 0–200 μM), or 2DG (0.6 mM) and carboplatin. Cell viability was assessed using a CellTiter cell proliferation assay kit (Promega), according to the instructions of the manufacturer. In brief, tetrazolium dye solution (20 μL) was added to each well, followed by incubation at 37 °C for 3 h, then measurement of absorbance at 490 nm, using a PHERAstar Omega plate reader (BMG Labtech, Mornington, Australia), as previously described [39]. Cell viability versus concentration of the test agent was plotted and used to determine the concentration causing a 50% reduction in cell viability (GI_50_).

### 4.5. Colony Formation Assay

The colony-forming ability of cell lines was assessed using a previously established method [40]. Cells (100,000/well) were seeded overnight in six well plates, followed by treatment for 72 h with 2DG (0.6 mM), carboplatin (GI_50_ concentration determined from cell viability assays), carboplatin, and 2DG, or PBS as a control, before non-enzymatic dissociation then replating at low density in a 24-well plate (500 cells/well), followed by growth for 1–2 weeks. The media was then removed from the plates and washed gently with PBS twice, before staining with 0.1% crystal violet (Sigma-Aldrich) in 2% ethanol. The stain was removed after 20 min and plates scanned using an Odyssey Imaging System and associated V3.0 software (LI-COR Biosciences, Millennium Science, Mulgrave, Australia) at 700 nm wavelength. Images were processed in ImageJ software, and the colony area and staining intensity quantified for each well using the ColonyArea ImageJ plugin, as described [41,42]. 

### 4.6. Fluorescence Microscopy Analysis

Glycogen content was examined qualitatively by fluorescence microscopy as previously described [31,43]. Briefly, cells were seeded into eight well chamber glass plates (Ibidi, DKSH, Victoria, Australia) and treated for 72 h with carboplatin (GI_50_ concentration determined from cell viability assays), carboplatin, and 2DG (0.6 mM), or PBS as a control. Cells were washed three times in warm PBS, and then incubated in a PBS solution containing green fluorescent 2-NBDG (300 µM) in the dark for 3 h, to permit monitoring of glucose uptake. Cells were then washed 3 times in PBS, followed by incubation in fresh warm media for 3 h. Hoechst dye was added 30 min prior to imaging to visualize nuclei. Cell staining was then imaged using a spinning-disk confocal imaging system incorporating a Nikon TI inverted microscope (Nikon, New York, NY, USA) equipped with a Borealis-modified Yokogawa CSU-X1 confocal head (Spectral Applied Research, ON, Canada) and a Clara cooled interline charge-coupled device (CCD) camera (Andor Technology, Belfast, UK).

### 4.7. Quantitative Glycogen Content Measurements

Relative glycogen level was assessed in cells treated for 72 h with carboplatin (GI_50_ concentration determined from cell viability assays), carboplatin and 2DG (0.6 mM), or PBS as a control. Glycogen levels were quantified as described previously using a kit from Megazyme (Deltagen, Victoria, Australia) [44]. Briefly, the D-glucose containing supernatant of amyloglucosidase treated cell lysates was incubated in a buffered solution containing adenosine triphosphate, NADP, glucose-6-phosphate dehydrogenase then hexokinase after an initial baseline reading (Roche). Controls included lysates unreacted with amyloglucosidase and deionized water. Absorbance at 340 nm was recorded for 30 min, and the concentration of NADPH, as an indirect stoichiometric measure of D-glucose concentration, was determined by extrapolation from a standard curve prepared from reactions using known concentrations of D-glucose as substrate. The results were normalized to protein content, determined using a bicinchoninic acid assay, then graphed as a relative measure of glycogen content.

### 4.8. Bioenergetic Analysis of Glycolysis and Mitochondrial Respiration

ECAR and OCR were quantified in real time using a Seahorse XFe Extracellular Flux Analyser (Seahorse Bioscience, Agilent, In Vitro Technologies, Noble Park North, Australia), as described previously [45,46]. Cells (10,000/well) were seeded into Poly-L-Lysine coated XF96 cell culture plates and allowed to adhere overnight in cell culture media. Cells were then treated for 24 h with PBS (control), carboplatin (GI_50_ concentration determined from cell viability assays), or carboplatin and 2DG (0.6 mM). On the day of the assay, the cells were washed twice with XF RPMI media (Seahorse Bioscience), then cultured in this media supplemented with 10 mM glucose, 2 mM glutamine, 5 mM HEPES, and 1 mM sodium pyruvate (pH adjusted to 7.4 + 0.05). Cells were then cultured in a non-CO2 incubator for 1 h prior to the start of the experiment, when rotenone and antimycin A (5 µM each) were injected into the media to eliminate cellular citric acid cycle activity, followed by 2DG (100 mM) to eliminate glycolytic activity. The assay plates included control blank wells containing only media to which the various reagents were added for experimental wells. Measurements from blanks were automatically subtracted by the instrument Wave 2.6 software. Data were normalized to the cell number per well, which was determined by imaging of DAPI stained cells using the Cytell Cell Imaging System (GE Healthcare, Parramatta, Australia). 

### 4.9. Assessment of the Effect of 2DG on the Efficacy of Carboplatin In Vivo

Mouse experiments were approved by the University of Queensland Animal Ethics Committee (approval 348/15 and 470/18), and research involving samples collected from human subjects was approved by the Mater Health Services Human Research Ethics Committee (project 29596). For xenografts of an OCC cell line female NOD.Cg-Prkdcscid Il2rgtm1Wjl/SzJ (NSG) mice (8–10 weeks old; The Jackson Laboratory, Bar Harbor, ME) were injected intraperitoneally (i.p.) with luciferase-labelled TOV21G cells (2 × 10^5^ cells/mouse) in PBS (200 μL). As measures of toxicity, mouse health was monitored by daily observation and weekly measurement of body weight. Tumor burden was qualitatively assessed regularly by peritoneal palpation, and quantitatively by weekly bioluminescence imaging, using an IVIS system and associated LivingImage software, as described previously [7,38]. After two weeks of tumor growth, mice were randomized into groups of five and treated i.p. with 2DG (50 mg/kg/week), carboplatin (10 mg/kg/week [47]), combined 2DG and carboplatin (at monotherapy dose) or PBS as a control. At the first signs of distress on day 37 after cell injections, all mice were euthanized. Ascites volume was measured and tumor nodules larger than 8 mm^3^ were counted [7].

Experiments involving patient-derived xenograft (PDX) models employed two previously described models PH138 and PH250, which were derived from stage 1C and recurrent tumors respectively [47], and model LP121, which was derived from a histologically confirmed stage 1B OCC tumor resected from a 48 year old patient at Mater Hospital, Brisbane, who received no adjuvant chemotherapy and was recurrence free 16 months after surgery. Thawed cell slurries from each PDX stored frozen at −196 °C were injected i.p. into 2–3 female 6–8-week-old NSG mice (equivalent to 0.2 g pelleted cell aggregate/mouse) in PBS (200 µL). When these mice were moribund due to disease burden, tumor nodules within the peritoneal cavity were collected and processed for propagation. Tumor tissue was mechanically disrupted in the absence of proteases and under sterile conditions by passing through a size 50 stainless steel wire mesh (Sigma-Aldrich). After centrifugation at low speed, the resulting cell pellet was resuspended in PBS then injected i.p. into mice or resuspended in a solution of FBS and 10% DMSO, then stored in liquid nitrogen. After two weeks of tumor growth mice were randomized into four groups of six and treated i.p. with 2DG (50 mg/kg twice weekly), carboplatin (1 mg/kg/week [39]), 2DG and carboplatin (at monotherapy dose), or PBS as a control. As measures of toxicity, mouse health was monitored by daily observation and weekly measurement of body weight. Tumor burden was qualitatively assessed regularly by peritoneal palpation. At the first signs of distress, all mice were euthanized and tumor nodules larger than 8 mm^3^ were counted [7]. For histological analysis, small portions of tumor tissue were fixed in formalin, embedded in paraffin, and hematoxylin, and eosin-stained sections assessed for clear cell histology by an anatomical pathologist (RL, JEA).

### 4.10. Statistical Analysis

In vitro assays were performed with at least three replicates on three independent occasions. Assay data were analyzed with an unpaired t-test with Šídák-Bonferroni correction, and results from in vitro and in vivo assays are presented, respectively, as mean ± SEM and mean ± SD, with *p* values ≤ 0.05 considered significant. Data and statistical analyses were performed using Prism 6.0 software (GraphPad, San Diego, CA, USA)

## 5. Conclusions

In summary, our results show for the first time that low levels of the small molecular weight disruptor of glucose utilization 2DG significantly improve the efficacy of carboplatin chemotherapy. Centres with high volumes of OCC cases could conduct clinical studies to evaluate whether this efficacy will extend to patients. Our results provide the rationale for histotype-specific treatment of OCC by targeting a prominent metabolic feature of this and other malignancies. Our findings also raise the possibility that low doses of other therapies that target glycogen metabolism could similarly improve chemotherapy efficacy for OCC and potentially other clear cell pathologies.
